# Near Infrared 45°/0° Reflectance Factor of Pressed Polytetrafluoroethylene (PTFE) Powder

**DOI:** 10.6028/jres.104.013

**Published:** 1999-04-01

**Authors:** Maria E. Nadal, P. Yvonne Barnes

**Affiliations:** National Institute of Standards and Technology, Gaithersburg, MD 20899-0001

**Keywords:** bidirectional reflectance distribution function (BRDF), diffuse reflectance, 45°/0° reflectance factor, pressed polytetrafluoroethylene (PTFE) powder

## Abstract

Pressed polytetrafluoroethylene (PTFE) powder is commonly used as a reflectance standard for bidirectional and hemispherical geometries. The wavelength dependence of the reflectance factor of PTFE is presented for the near-infrared spectral region (800 nm to 1600 nm) for the 45°/0° geometry, as well as in the visible spectral region (380 nm to 800 nm) for comparison with previously published results.

## 1. Introduction

The perfectly reflecting diffuser is defined as a Lambertian diffuser with a reflectance factor equal to unity. While no real material has such ideal properties, there is a compelling need by national laboratories, standards organizations, optical material industries, and instrument manufacturers for a reflectance standard that approximates the ideal. Such a reflectance standard should have a reflectance factor close to unity over the ultraviolet to infrared spectral region, have very diffuse reflectance properties, be highly opaque, have no fluorescent excitation for wavelengths longer than 300 nm, and be spatially uniform, cleanable, durable, and stable. Materials such as polytetrafluoroethylene (PTFE) and barium sulfate (BaSO_4_) are close approximations of ideal diffusers and are thus widely used as reflectance standards.

The Optical Technology Division of the National Institute of Standards and Technology (NIST) has determined the average reflectance factor of pressed PTFE powder for the 6°/hemispherical geometry over the spectral region of 200 nm to 2500 nm [1,2] and for the 45°/0° geometry over the spectral region of 380 nm to 770 nm [3]. The 45°/0° reflectance factor of pressed Algo-Flon (F5) PTFE powder^1^ over the spectral range of 380 nm to 1600 nm is presented here. These measurements are intended to show the general trend of the wavelength dependence of PTFE for the 45°/0° reflectance factor in the near-infrared (NIR) spectral region. This paper does not provide calibrated reflectance factors because of the variability of PTFE, shown in Ref. [3].

## 2. Theory

The reflectance factor of a sample is defined [4] as the ratio of the radiant flux reflected in the direction delimited by a given cone, with the apex at the surface of the sample, to the radiant flux reflected in the same direction by a perfectly reflecting diffuser illuminated in the same manner. The 45°/0° reflectance factor is measured at an incident angle of 45° and a viewing angle of 0°.

A detailed derivation of the measurement equations is given in Refs. [5,6]. Reflectance factor *R* is defined in terms of the bidirectional reflectance distribution function (BRDF) *f*_r_ as

$$R=\text{π}\cdot f_{r}.$$ (1)

The BRDF is the ratio of the reflected radiance at a given angle to the incident irradiance at another given angle, both angles being measured from the normal to the sample surface. For a fixed linear polarization of the incident beam and incident and viewing directions in the same plane, the measurement equation for BRDF is given by

$$f_{r}\left(\theta_{i},\theta_{v},\lambda ,\sigma \right)=\frac{S_{r}\left(\theta_{i},\theta_{v},\lambda ,\sigma \right)}{S_{i}\left(\lambda ,\sigma \right)}\cdot \frac{D^{2}}{A_{r}\cdot \cos\theta_{v}},$$ (2)

where *θ*_i_ and *θ*_v_ are the incident and viewing angles, respectively, *λ* is the wavelength, *σ* is the polarization, *S*_i_ and *S*_r_ are the signals from the incident and reflected radiant flux, respectively, *D* is the distance from the sample to the aperture stop of the detector, and *A* is the area of the aperture stop.

Calculation of the reflectance factor requires measurements of the incident and reflected radiant flux. To compensate for instrumental drift, the reflected signal measurements are sandwiched between measurements of the incident flux after correcting for dark signals. The BRDF of unpolarized light is given by the average BRDF obtained from measurements with perpendicular and parallel polarization with respect to the plane of incidence.

## 3. Instrumentation

**Fig. 1:**
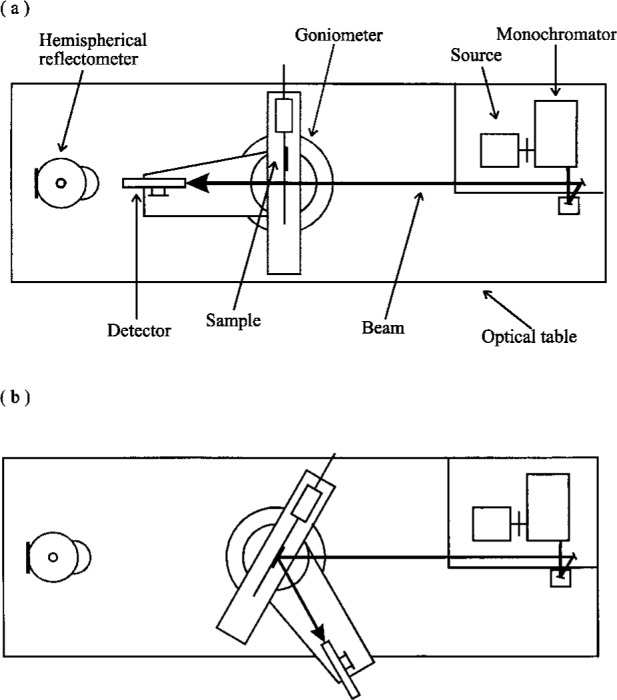
Schematic diagram of STARR with all the major components labeled, (a) depicts the incident flux measurement and (b) depicts the reflected flux measurement for the 45°/0° geometry.

The absolute spectral measurements of bidirectional diffuse reflectance were performed using the Spectral Tri-function Automated Reference Reflectometer (STARR) at NIST. Details of this instrument are given in Refs. [6,7]. A schematic diagram of the instrument setup with all the major components labeled is shown in Fig. 1.

The major components of the instrument are a source, a sample holder on a goniometer, and a detector. The source system provides a collimated, monochromatic beam of light with a known polarization over the spectral region of 200 nm to 2500 nm. The goniometer is a monoplane gonio-instrument with a rotating sample table and a movable detector arm. The sample table and the detector arm can rotate at a constant distance independently of each other but normal to the plane of incidence. The goniometer varies the angle of incidence from 0° to 80°, and the viewing angle can be any value greater than 5° relative to the incident beam. The distance from the sample axis of rotation to the aperture stop is 672.6 mm. The diameter of this aperture is 31.85 mm. The absolute reflectance is calculated from measurements of the incident and the reflected radiant fluxes obtain by rotating the detector arm. Two detectors were used for the measurements described in this paper. A UV-enhanced silicon photodiode is used for the spectral region of 380 nm to 1000 nm and a germanium photodiode, thermoelectrically cooled to −20 °C mounted on a 38 mm integrating sphere coated with PTFE, is used for the spectral region of 900 nm to 1600 nm. The output photocurrent is measured by a picoammeter and the value is sent to a computer. This computer controls all of the goniometer motion and data acquisition processes.

## 4. Results and Uncertainties

Following the procedure described in Ref. [3], four fresh samples of pressed PTFE using Algo-Flon (F5) type powder were prepared. The samples were 10 mm thick to avoid translucency in the material. Measurements were taken before and after the surfaces of the samples were imprinted with a rough surface using 180-grit sandpaper. This imprinting was done to more fully randomize the surface. The sandpaper was laid down on the surface and pressed with minimal downward pressure to avoid any lateral motion.

The geometrical dependence was checked by measuring the reflectance factor for the 45°/0° and 0°/45° geometries for samples before and after sandpaper imprinting. According to the Helmholtz reciprocity principle, the 45°/0° and 0°/45° reflectance factor measurements should agree with each other to within the instrument noise. This test checks the spatial uniformity of the surface of PTFE samples. The measurements performed on both types of surfaces for both geometries agree to within the overall uncertainty of the instrument.

**Fig. 2:**
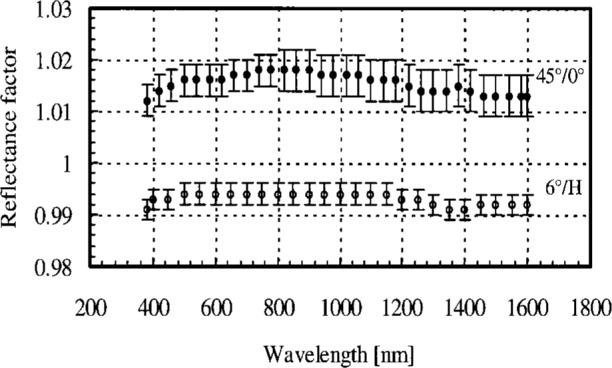
Reflectance factor as a function of wavelength for pressed PTFE Algo-Flon (F5) powder for 45°/0° (filled circles) and 6°/hemispherical (open circles) geometries.

A plot of the 45°/0 and 6°/hemispherical [2] reflectance factors as a function of wavelength is shown in Fig. 2. Following the procedure for uncertainty analysis presented in Ref. [6], the relative expanded uncertainty (*k* = 2) for the 45°/0° reflectance factor of pressed PTFE powder is 0.3 % at 600 nm and 0.4 % at 1200 nm. The components of uncertainty are divided into those arising from random and systematic effects. The random effects, which include the source stability, incident and reflected signal, and sample, result in a relative expanded uncertainty of 0.1 % and 0.2 % for the visible and NIR spectral regions, respectively. The systematic effects, which include the wavelength uncertainty, stray light, angle of reflection, aperture stop area, and the distance from the sample to the aperture stop, result in a relative expanded uncertainty of 0.3 % for both spectral regions. The 45°/0° reflectance factor for the visible region presented here are slightly larger, but within the uncertainty limits, of previous studies on PTFE [3,7]. The 45°/0° reflectance factor of PTFE strongly depends on batch to batch variations as shown in [3]. The largest variations are observed for the shortest wavelengths. The reflectance factor has a slight dependence on wavelength over the measured spectral region and is greater than one. Previous measurements of PTFE [6] have shown that the reflectance factor depends on both the incident and observation angles, and can be greater than one, particularly toward large specular observation angles. This result illustrates that a single reflectance measurement for a pair of incident-reflected angles is not sufficient to estimate the total integrated scattered light from a sample.

## 5. Conclusions

The wavelength dependence of the 45°/0° reflectance factor of pressed PTFE for the 380 nm to 1600 nm spectral region was investigated. The measurements for the visible region demonstrate the reproducibility of the 45°/0° reflectance scale, while the NIR measurements extent the region of published values. The reflectance factor in the near-infrared spectral region of 800 nm to 1600 nm has a slight spectral dependence, as is also the case in the visible region. The signal to noise ratio (SNR) in the near-infrared region is on the average two orders of magnitude smaller than in the visible region, suggesting improvement possibilities in the instrument. A new InSb detector is currently being modified at NIST to be added to the capabilities of STARR. This new addition will improve the SNR and extend the infrared measurement capabilities from 800 nm to 2500 nm.

## Footnotes

1 Certain commercial equipment, instruments, or materials are identified in this paper to foster understanding. Such identification does not imply recommendation or endorsement by the National Institute of Standards and Technology, nor does it imply that the materials or equipment identified are necessarily the best available for the purpose.
